# Towards robust diagnosis of COVID-19 using vision self-attention transformer

**DOI:** 10.1038/s41598-022-13039-x

**Published:** 2022-05-26

**Authors:** Fozia Mehboob, Abdul Rauf, Richard Jiang, Abdul Khader Jilani Saudagar, Khalid Mahmood Malik, Muhammad Badruddin Khan, Mozaherul Hoque Abdul Hasnat, Abdullah AlTameem, Mohammed AlKhathami

**Affiliations:** 1 Knightec AB, Vasteras, Sweden; 2 LIRA Center, Lancaster University, Lancaster, LA1 4YW UK; 3 Department of Computer Science and Engineering, Oakland University, Rochester, MI USA; 4 Information Systems Department, College of Computer and Information Sciences, Imam Mohammad Ibn Saud Islamic University (IMSIU), Riyadh, Saudi Arabia

**Keywords:** Infectious diseases, Machine learning, Diagnosis

## Abstract

The outbreak of COVID-19, since its appearance, has affected about 200 countries and endangered millions of lives. COVID-19 is extremely contagious disease, and it can quickly incapacitate the healthcare systems if infected cases are not handled timely. Several Conventional Neural Networks (CNN) based techniques have been developed to diagnose the COVID-19. These techniques require a large, labelled dataset to train the algorithm fully, but there are not too many labelled datasets. To mitigate this problem and facilitate the diagnosis of COVID-19, we developed a self-attention transformer-based approach having self-attention mechanism using CT slices. The architecture of transformer can exploit the ample unlabelled datasets using pre-training. The paper aims to compare the performances of self-attention transformer-based approach with CNN and Ensemble classifiers for diagnosis of COVID-19 using binary Severe Acute Respiratory Syndrome Coronavirus 2 (SARS-CoV-2) infection and multi-class Hybrid-learning for UnbiaSed predicTion of COVID-19 (HUST-19) CT scan dataset. To perform this comparison, we have tested Deep learning-based classifiers and ensemble classifiers with proposed approach using CT scan images. Proposed approach is more effective in detection of COVID-19 with an accuracy of 99.7% on multi-class HUST-19, whereas 98% on binary class SARS-CoV-2 dataset. Cross corpus evaluation achieves accuracy of 93% by training the model with Hust19 dataset and testing using Brazilian COVID dataset.

## Introduction

The coronavirus disease (COVID-19) has emerged as one of the deadliest virus of the modern times, and so far, has resulted in over 5.56 million deaths worldwide^1^. Due to its very contagious nature, earlier screening of the infection has proven to be essential to reduce or minimize the further prevalence of COVID-19 disease. Various methods have been utilized to diagnose this infectious disease. Real Time—Polymerase Chain Reaction (RT-PCR) tests are used to confirm the diagnosis, but it is resource and time consuming^2^. Besides this, RT-PCR tests has higher false negative values that can cause intense consequences. Several imaging techniques such as Computed Tomography (CT), and Chest X-rays (CXR) are employed to examine the infected COVID-19 patients. These techniques assist the clinicians distinguishing the Covid-19 effects on different organs. However, research findings suggests that chest CT is better at correctly diagnosing COVID-19 infection and has low rate of missed diagnoses^3^. CT slices has been considered as essential tool for screening of COVID-19 screening as its able to perform fast prediction as compared to RT-PCR test. In particular, several researchers have shown that CT scan is considerably helpful in identification of COVID-19 infection. In addition, visual analysis of CT images is also time-consuming, especially with large number of patients and in large studies^4^. Moreover, certain abnormalities in chest CT images provides the indication of COVID-19 infection such as ground-glass opacities and consolidation.

CT imaging has been considered as vital substitute and effective way for screening of COVID-19. CT imaging can do fast prediction as compared to RT-PCR^4^. Existing research revealed that CT images comprises of potential signs of infection, but infection could be not related to COVID-19. That implies challenges for radiologists in identifying COVID-19 infections by visually analysing the CT images^4^. Moreover, visually analysing the CT images is also time-consuming with large number of patients. With the urgent need for diagnosis of COVID-19 pandemic, several existing studies have used vision transformer^5^ or deep learning-based techniques for COVID-19 diagnosis using CT scan images^6–9^, but they suffered from poor generalization capability. To mitigate this issue, most used measures are to build model with countless training data^10^ but constructing large scale label dataset is difficult task. Most of the previous research studies employ CNN models. Although convolutional neural network architecture has shown outstanding performance in computer vision tasks, it may not be optimal for disease classification due to difficulty in selection of an optimal CNN architecture^11^. To enhance the local related features, self-attention module performs feature recalibration which indirectly reduces the role of the features at the spatial and channel levels^12^.

In addition, one of the main problems in the computer vision area was that integrating the global relationship among the pixels are required by convolutional neural networks. To overcome this limitation, vision transformer was proposed which used self-attention mechanism for modelling the pixel dependency among pixels^13^.

In this paper, a self-attention transformer-based approach has been developed to accurately diagnose the COVID-19 using CT scan images. Vision Transformer (ViT) based approach model the long-range dependency between the pixels using self-attention mechanism and showed SOTA performance in image classification. In addition, proposed approach addresses the problem of generalization capability on unseen data.

In summary, main contributions of our research work are as follows.

- A self-attention transformer-based model for diagnosis of COVID-19 is proposed.
- We experimentally demonstrated that proposed approach outperforms other CNN based models as well as ensemble classifiers especially in terms of the generalization on unseen data.

The remainder of paper is structured as follow. “Literature review” summarizes the existing literature. “Proposed ViT-based method for COVID-19 diagnosis” describes the proposed framework. Dataset description and Experimental results are presented in “Experiments”. “Discussion” discusses the rationale behind the results of the experimentation, and finally, we conclude this research work in “Conclusion”.

## Literature review

Digital technologies have assisted the scientists to counter the COVID-19 epidemic from different perspectives. Many different techniques have been developed in this regard. Haleem et al.^14^ has described the effects of COVID-19 in daily life and listed its impacts on health systems specifically. Haleem et.al has detailed the significant applications of big data in COVID-19 pandemic in^15^, where they have listed the apps from travel history to the identification of COVID-19 cases. Another aspect of the epidemic has been studied by Suman et al.^16^.

Transformer^17^, a deep neural network mechanism was originally designed for natural language processing tasks. Self-attention mechanism of transformers assists long range dependencies. In computer vision area, the application of Transformer has become an active investigation area, results in outstanding performance in several computer vision tasks. Vision Transformer (ViT) was applied for the first time to analyze the image in^5^ and results were so good that convolutional operation was replaced with ViT. Consequently, authors also designed a hybrid architecture by combining transformer to the Resnet backbone of convolutional neural network. Transformer can primarily focus on modeling global attention using Resnet extracted features. Results achieved from experiments imply that hybrid approach can be able to produce high performance with less computational resources. Transformer application in computer vision have become active area of research, results in various models of ViT in a variety of computer vision tasks such as object detection^18^, classification^19^, segmentation^20^. Authors in^19^, have claimed that CNN dependency is no more a necessary condition, and they have validated it through direct application of transformer to the sequence of images.

Furthermore, ViT based model^5^ was developed for diagnosis and severity measurement of COVID-19 using CXR disease. Several datasets including Brixia dataset, CNUH, YNU, KNUH datasets containing CXR images were used for performance evaluation of this model which showed outstanding performance as compared to CNN based models achieving 86.9% accuracy. However, ViT based model^5^ did not perform cross corpus evaluation. Whereas the proposed self-attention transformer performs cross corpus evaluation results in higher accuracy than existing methods. Severity prediction proposed in^5^ can be of greater importance in circumstances where the experienced staff or the examination infrastructure is not available due to any reason.

Inspired by the classical non-local means method in computer vision, Wang X et.al have presented non-local operations as a generic family of building blocks for capturing long-range dependencies^21^. They are using a weighted sum of the features at all positions to determine the response at a position. Another latest effort reported on using 10 pretrained Convolutional Neural Network models for COVID-19 CT scans classification^22^. This study stated that Xception and ResNet-101 delivered best classification accuracy on CT dataset training and testing. ResNet-101 can be used to characterize and diagnose COVID-19 infections with substantial cost. Additional earlier work on COVID-19 CT scans classification were reported in several studies such as in^6–8^. A 3D deep NN known as COVNet was designed for recognition of COVID-19 from chest CT scans^6^ where authors have suggested to use the multidisciplinary approach as they consider it not possible to differentiate all lung diseases based simply on the imaging appearance on chest CT^6^. Classification study^7^ uses a small training data from scan from patients with severe disease level and thus performs not very well. Contrary to^7^, the deep learning models established in^8^ were effective at the earlier stage of the disease. Problem with^9^ is that it considers only 2 class classification as the 3 class classification data is either very limited or not available for public use.

Proposed model was built on pretrained RestNet50. Both 2D and 3D features were extracted by network from CT scans. Researchers conducted study^9^ on classification of COVID-19 using 16 pretrained CNNs models. A large dataset of CT scans was collected for the experimental purpose. These pretrained CNN models were trained on ImageNet database images. Amongst the 16 CNNs models, DenseNet‐201 achieves high accuracy, sensitivity and specificity value and area under curve. Moreover, transfer learning with whole image slices and without data augmentation delivered better classification accuracy than the using data augmentation. In case of training using data augmentation, DenseNet-201, ResNet-18, ShuffleNet, MobileNet-v2 gives the average accuracy of above 95%, however DenseNet-201 attains overall highest accuracy of 96.20%. GoogLeNet, ResNet-18, ShuffleNet, MobileNet-v2, ResNet-101, ResNet-50, DenseNet-201, and Inception-v3 results in average sensitivity above 95%, whereas ResNet-18 achieves average sensitivity of 98.99%. A semi supervised neural network model^23^ was proposed which comprises of PQIS-Net for lung CT images segmentation. Proposed model was evaluated on publicly available dataset of Brazilian data set and IEEE CCAP data set. Segmentation performance of proposed PQIS-Net, 3D-Unet and ResNet50 on these datasets was measured using Dice Similarity (DS). It has been observed that, proposed model performs best in patch-based classification having FC layer. The accuracy achieved by model^23^ was like ResNet50 whereas precision was like 3D-Unet. It was shown that model performs better than 3D-Unet in terms of recall, accuracy, and F1-score on the Brazilian data set. Although experimental results reveal that 3D-Unet and ResNet50 slightly outperform than their proposed model^23^ in segmentation task.

Success of transformers in computer vision is extraordinary and particularly when using the large-scale datasets in vision applications. Use of transformer vision in medical imaging and specifically in image classification is a relatively new and evolving area and in comparison, to the natural images. The challenge in medical images for ViT comes forward in form of long-range dependencies and multi-modality. Matsoukas C has put up a good case for transformer in their study titled "Is it Time to Replace CNNs with Transformers for Medical Images?"^24^. They have shown that even if the datasets are smaller, ViT can achieve the same performance level with the help of transfer learning and as dataset gets grow, performance of ViT becomes better. Shao C has used transformer for whole slide image classification. Shao work uses the transformer based Correlated Multiple Instance Learning for this purpose^25^. Proposed model achieves the better performance, faster convergence and clinical interpretability, vital for the corelated information analysis. Proposed algorithmic model, TransMIL network is easy to train and has applicability on different sort of data (balanced or non-balanced) for binary or multiple classification. Shen Z has used Convolution in Transformer Network for End-to-End Polyp Detection^26^. Proposed model COTR produces the results which are quite comparable to the existing state-of-the-art methods but however this produces low confidences when it encountered sessile polyps. For 3D MRI analysis, Jun E has used medical transformer using Universal Brain Encoder^27^. Results from their experiments show that transformer takes into consideration the relations over distant slices and thus captures volumetric features. Dai Y has proposed an architecture name TransMed based on the Multi-modal Medical Image Classification^28^. Proposed TransMed is easy to implement and has a flexible structure, but it is not pure transformer structure. Pure transformer can enhance the results as it is evident from the results shown by different researchers in based on large-scale natural image datasets.

Deep learning-based algorithm was designed to predict the COVID-19 in^29^. To test the proposed algorithm, HUST19 dataset was used which reveals that the algorithm achieved area under the roc curve of 0.944. Another publicly available dataset named COVID-19-dataset was utilized to test the performance of transfer learning based COVID-19 diagnosis approach^30^. Their proposed approach integrates transfer learning with supervised learning to avoid over-fitting problem. The approach achieves AUC of 94% in detecting COVID-19 from CT slices. A deep learning-based system was developed^31^ to detect COVID-19 using 3D CT scans. They collect their own dataset to test the proposed approach. Performance evaluation on CT scan dataset showed that proposed approach obtained accuracy of 90% and AUC 95% respectively.

## Proposed ViT-based method for COVID-19 diagnosis

Since 2012, convolutional neural networks (CNN) have become widely used model for computer vision tasks. The major advantage CNN provides in comparison to existing image classification algorithms is automated learning of its network to optimize the filters, making it independent from human intervention. However, CNN architecture is domain specific and can take more computational time. As CNN utilize the pixel information where each pixel illustrates different importance for target task that cause repetition in representation and computation. Furthermore, CNN do not interpret features structural dependency.

Looking forward to salable vision models, computationally efficient and more domain agnostic architectures is necessarily to achieve state-of-the-art results. Vision Transformer (ViT), a vision model based is a first step in this direction, originally planned for NLP tasks. ViT demonstrate an input image as number of image patches, analogous to sequence of word embedding utilized when Transformers is applied to text and predicts class labels of image directly. With an adequate training, computational cost for Vision Transformer is optimal in comparison to the CNN^32^.

**Figure 1:**
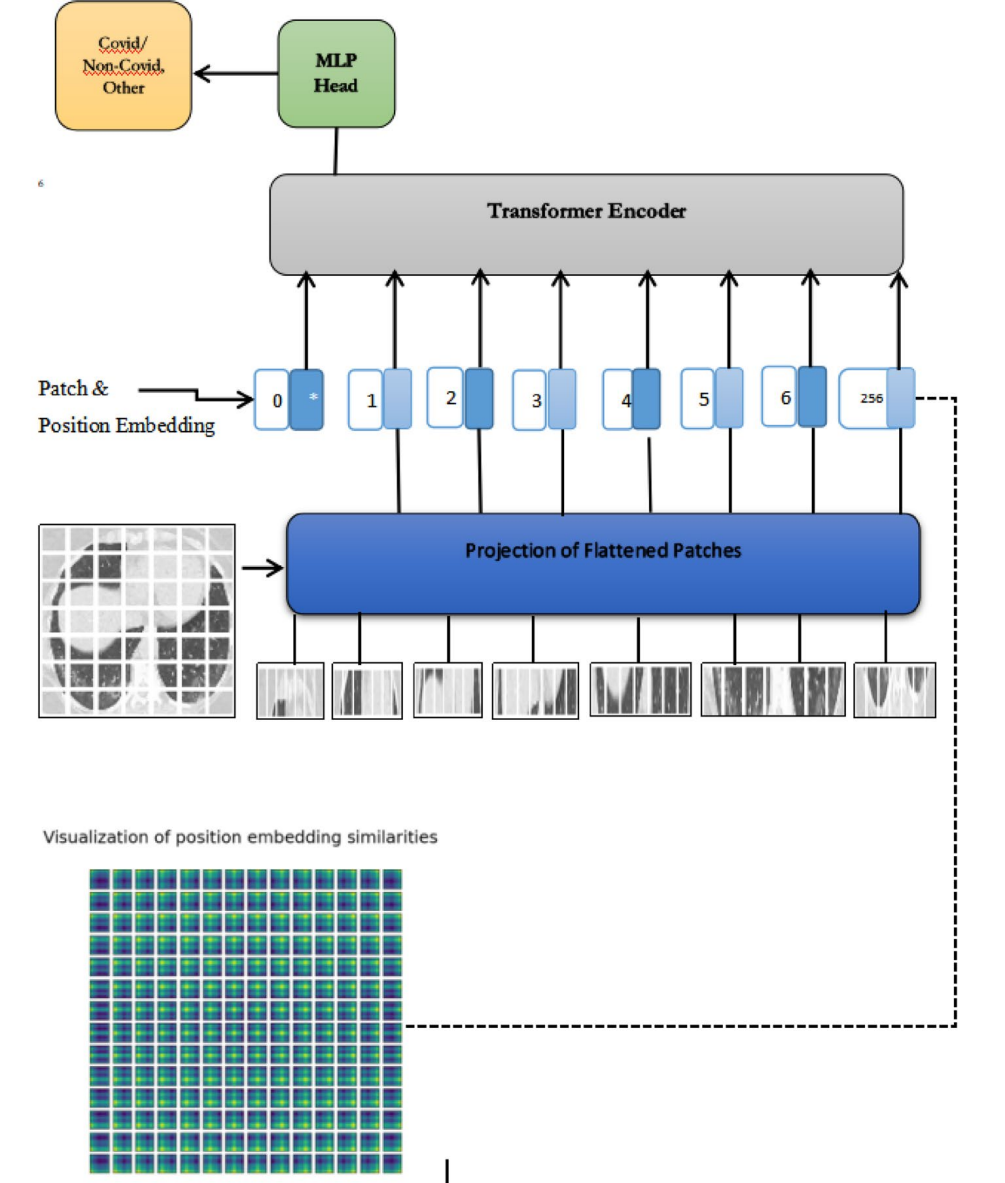
Overview of proposed approach.

**Figure 2:**
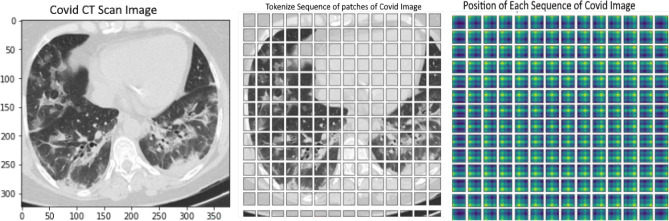
(**a**) COVID CT scan image, (**b**) number of patches (**c**) positional embeddings.

Figure 1 represents the workflow of the proposed approach. Input image is divided into number of fixed patches. These patches are flattened, and positional embedding are assigned to given it to transformer encoder. Classification is performed using multi-layer perceptron head in transformer encoder. Transformer usage allows more elaborated and consistent predictions as compared to convolutional neural network. In the proposed approach, segmentation of image is performed using transformer encoder/decoder architecture which maps the sequences of patch embeddings to pixel level annotations as shown in Fig. 2.

An image x ∈ IH*W*C is divided into several patches x = [x_1_, …, x_n_ ] ∈ I. Each patch of image is flattened into a vector and linear projection of these flattened patches is performed to generate a sequence of patch embeddings x_0_ = [Ex_1_, …, Ex_n_] ∈ I^N×D^ where N represents the number of patches as shown in Fig. 2b. To capture the positional information, positional embeddings are added up to the sequence of patches for having a tokenize input sequence as represented in Fig. 2c.

**Figure 3:**
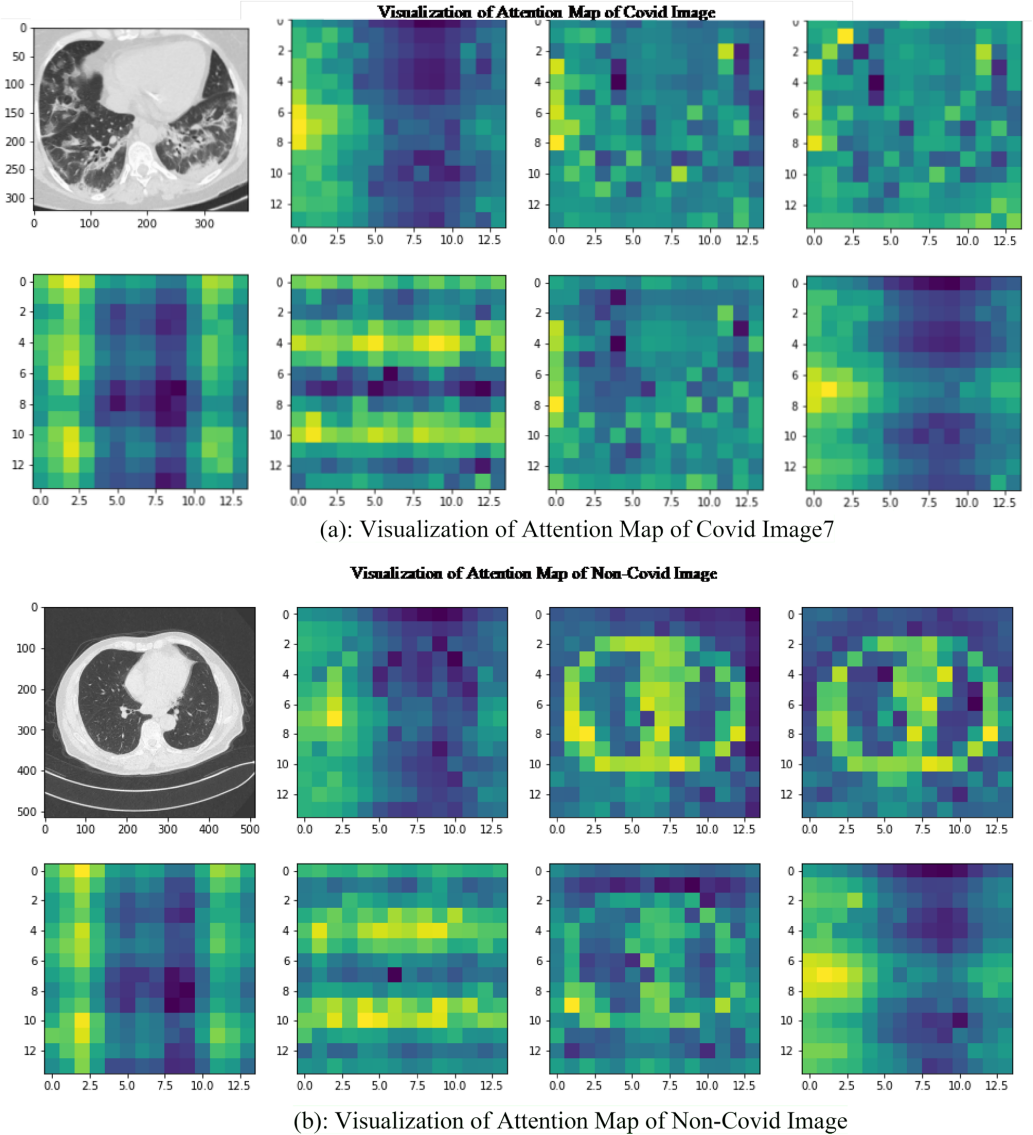
(**a**) Visualization of attention map of COVID Image7. (**b**) Visualization of attention map of non-COVID image.

A transformer encoder is applied to this sequence of tokens to produce a contextualized encoding that contains rich semantic information. The encoder layers of Transformer used in the proposed model is same as encoder of standard Transformer comprising of layer normalization, multi-layer perceptron, multi-head self_attention and residual connections. The self-attention mechanism comprises of three linear layers which maps the tokens into intermediate representations, keys, queries, and values. For interpret-ability of proposed model, attention map visualizations are generated for self-attention Transformer as shown in Fig. 3. The attention map of COVID and non-COVID image is visualized in different layers as shown in Fig. 3a,b. It illustrates the region of images which are significantly important relevance to machine translation using attention mechanism.

Small and larger models are considered for building the self-attention transformer. Parameters varying in encoder are the patch size, number of layers, input image size and number of epochs. We consider input image of different resolutions and use patch size of 8 × 8, 10 × 10 and 14 × 14. We compared the input CT scan image of size 300 × 300 and 150 × 150 with same experimental settings, as increasing the resolution of input image improves the performance of the model. We have used the image size of 72 and 100 and number of transformer layers employed are 8–10 in our experimentation. The transformer encoder sends out the packages to the attention layer that split the input into multiple heads and each head learn the self-attention mechanism. All the head's outputs are concatenated to passed to multi-layer perceptron and size of multi-layer perceptron used is [2048, 1024]. Layer normalization is implemented with skip connection in every block with epsilon value 1 − e6. We apply the dropout rate of 0.1 to regularize our model and data augmentation are performed using image flipping (horizontal), resizing (image size) and rotation (factor = 0.02). MLP head, a classification module is employed at the end which output the number of classes.

## Experiments

During the current COVID-19 pandemic, the availability of CT scan datasets is necessary and significant to provide deepen understanding and valuable information about this viral infection. It is essential for earlier diagnosis of COVID-19 and timely medical intervention. To perform experiment, 80% of dataset is chosen for training whereas 20% is assigned for testing purpose.

### Experimental setup and dataset

This section illustrates the two datasets considered for experimentation purpose. Datasets chosen for experimentation purpose have no missing values, up to date and accurate. Both datasets are open-source and are available.

#### Brazilian dataset (SARS-COV2 CT scan dataset)

**Figure 4:**
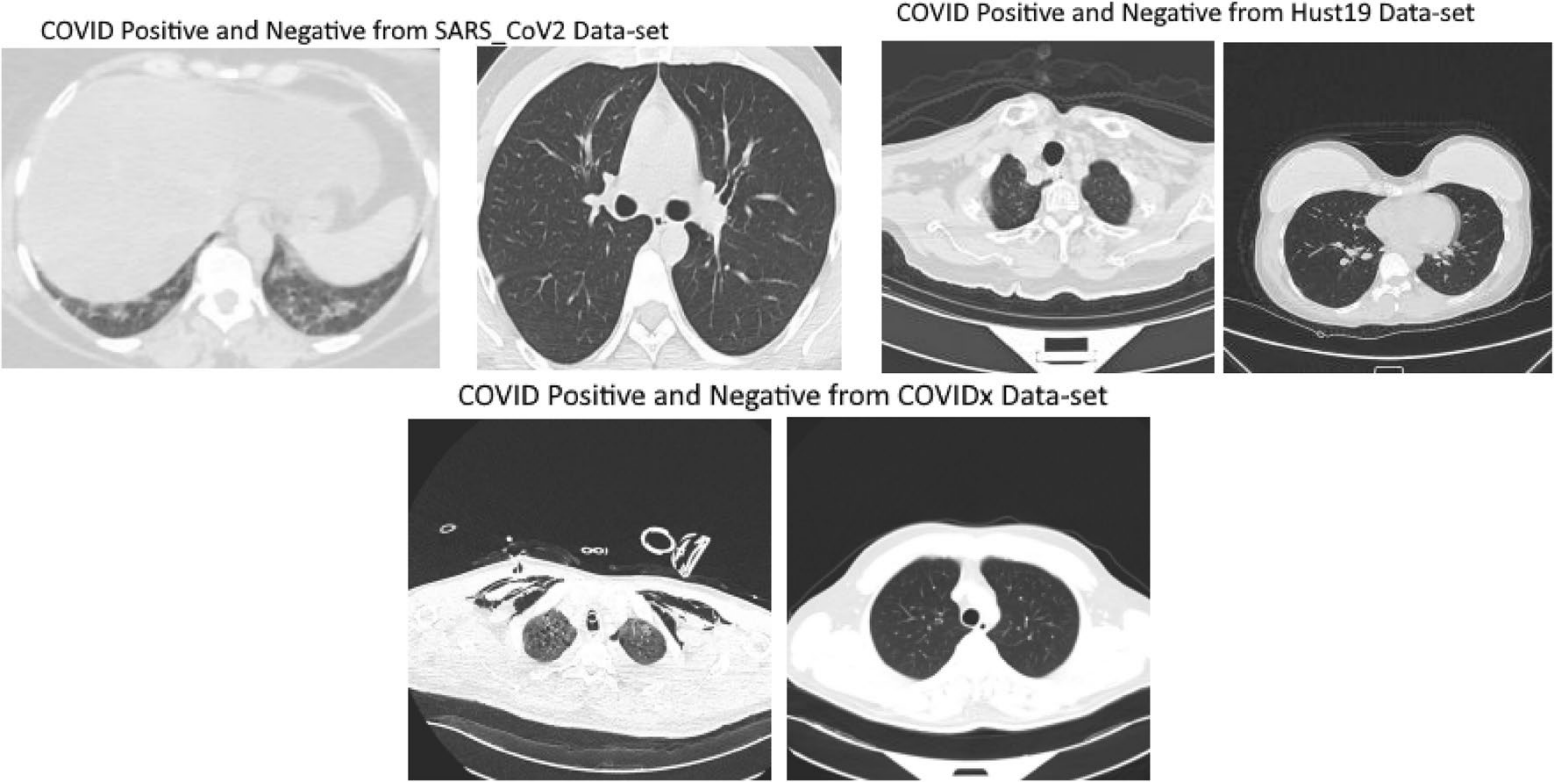
(1): examples of 3D CT scan images that are COVID positive (Left) and (2) non-COVID-19 (Right) from SARS_CoV2 Data-set. (3)Examples of 3D CT scan images that are COVID positive (Left) and (4) non-COVID-19 (Right) from Hust19 Data-set. (5) Examples of 3D CT scan images that are COVID positive (Left) and (6) non-COVID-19 (Right) from COVIDx Data-set.

The SARS CT scan dataset comprises of 2482 CT scans of 120 patients which includes 1252 CT scans of 60 infected patients and 1230 CT images of 60 patients which were non-infected shown in Fig. 4. The data was gathered from real patients from hospital in Sao-Paulo, Brazil hospitals.

CT Scan Images varies in image size such as image of small size consist of 104 × 153 dimension and larger image comprises of 484 × 416 dimension. The dataset contains heterogenous CT scan images having low number of instances. In additions, CT scan images are of different contrast and resolution. This open source dataset is available on https://www.kaggle.com/plameneduardo/sarscov2-ctscan-dataset. Basic purpose of this dataset was to promote development and research of artificial intelligent methods that are able to determine person infected by SARS-CoV-2 using CT scans. In case of SARS-CoV-2 CT-scan dataset (Brazilian dataset) necessary IRB and/or ethics committee approvals was obtained.

#### Hust19 dataset

Hust19 is multiclass dataset comprises of three types of CT slices such as Non_informative CT (NiCT), Postive CT (pCT) and Negative Ct (nCT). NiCT contains 5705 scan images which have no information about lung parenchyma whereas pCT includes 4001 CT scan images that contains imaging features related to COVID-19 pneumonia shown in Fig. 4. The third type of Hust19 dataset, negative (nCT) comprises of 9979 CT images which were not related to COVID19 pneumonia. The HUST19 CT scan data-set comprises of 19,685 CT scan slices. Dataset is open source and is available on https://bioengineeringcommunity.nature.com/posts/hust-19-for-predicting-covid-19-clinical-outcomes. Hust19 was made available under a CC BY-NC 4.0 license. Hust19 was accumulated from lab of union hospital, Wuhan. It contains number of chest CT (computed tomography) images and clinical features from patients having or without COVID-19.

### Comparison with state-of-the-art methods

**Figure 5:**
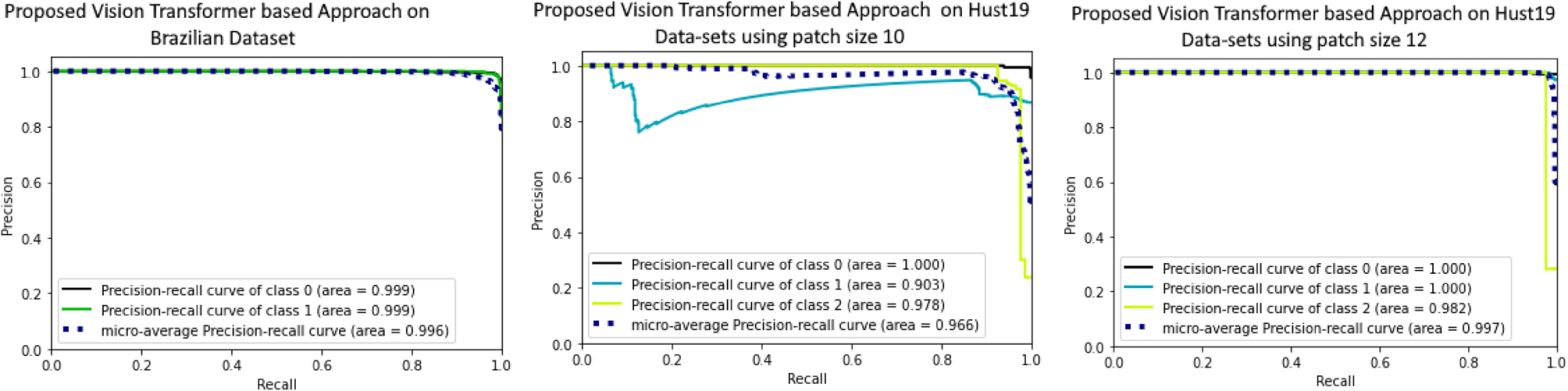
Performance evaluation of proposed vision transformer based approach (**a**): results on Brazilian dataset (**b**) results on Hust19 Data-sets using patch size 10 (**c**) results on Hust19 Data-sets using patch size 12.

The performance of proposed method is evaluated by comparing it with existing methods. Details on the performance evaluation of Brazilian Datasets are presented in Fig. 5a. To evaluate the proposed method performance, 80% of dataset is used for training while 20% is hold out for testing purpose. Several parameters are considered for experimental evaluation. Patch size of 10 × 10, 200 number of epochs and input image resolution of 300 × 300 was considered for comparing the proposed method with state of art methods. By increasing the input image size, patch size and resolution of image on HUST19 dataset, results in increase in accuracy of our approach as shown in Fig. 5b,c. Whereas, number of epochs have no significant effect on proposed approach accuracy. Figure 5b reveals that when patch size of 10 is employed, AUC value is 0.966 whereas AUC tends to increase to 0.977 when patch size of 12 is used as shown in Fig. 5c.

Different methods are utilized for comparison propose such as VGG16, Inceptionv3 and Resnet50. We train the network for 200 epochs, and network is tested with test set in each epoch. There are 12 number of steps in each epoch. All the methods are evaluated with accuracy metric which allows us to make performance comparison with two CT scan images datasets.

**Figure 6:**
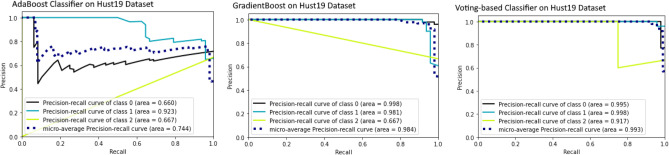
Performance evaluation of ensemble classifier on Hust19 Data-set (**a**) AdaBoost (**b**) GradientBoost (**c**) voting-based classifier.

**Figure 7:**
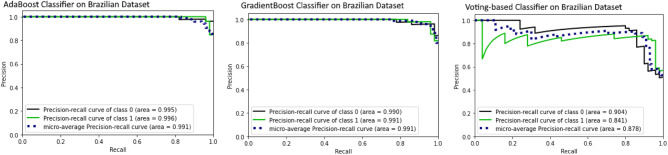
Performance evaluation of ensemble classifier on Brazilian Data-set (**a**) AdaBoost (**b**) GradientBoost (**c**) voting-based classifier.

For binary classification (COVID, Non-COVID) on Brazilian Dataset, the test accuracy of Resnet50, InceptionV3, VGG16 is 90.0%, 82.0%, and 81.0%, respectively. AUROC of three ensemble classifiers are shown in Figs. 6 and 7. These classifiers are applied on Brazilian and Hust19 dataset that shows better performance on Brazilian Dataset. However, AUROC of GradientBoost and voting based classifier reveals good performance in case of Hust19 dataset as well. Ada-boost, Gradient-boosting, voting classifiers achieved 84%, 96.0%, and 97.0% accuracy on Hust19 dataset respectively. It has been revealed from experimentation that loss value using the proposed ViT based approach is less as compared to state of art methods. The precision recall value on different classes of Hust19 dataset are shown in Fig. 5b revealing model good performance on two classes. Less precision and recall value of class 2 could be because of a smaller number of instances were available to train the model. Furthermore, resultant accuracy on hust19 dataset is 99.6%. The number of transformer layers, patch size and image resolution have an impact on precision recall curve. Proposed approach achieved 94% accuracy by using the image resolution of (150 × 150) and patch size of 6. We trained the models with 200 epochs, learning rate of (1 × e − 3), batch size of 156, patch size of 10, transformer layers of 8 and image resolution was set to (300 × 300) results in 98% accuracy on Brazilian and 99.6% accuracy on Hust19 dataset.

**Figure 8:**
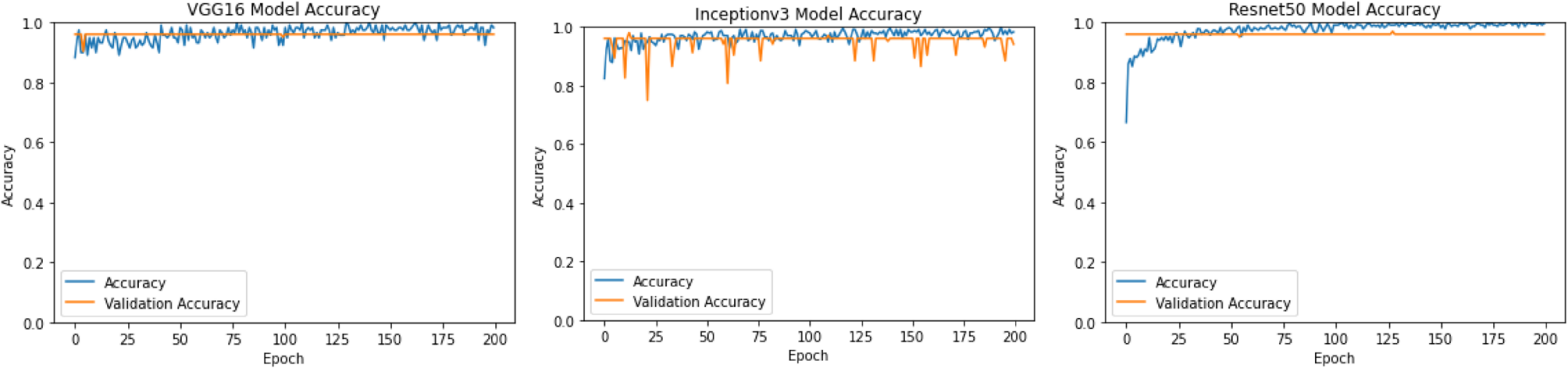
Performance evaluation of deep learning classifier on Hust19 Data-set.

**Figure 9:**
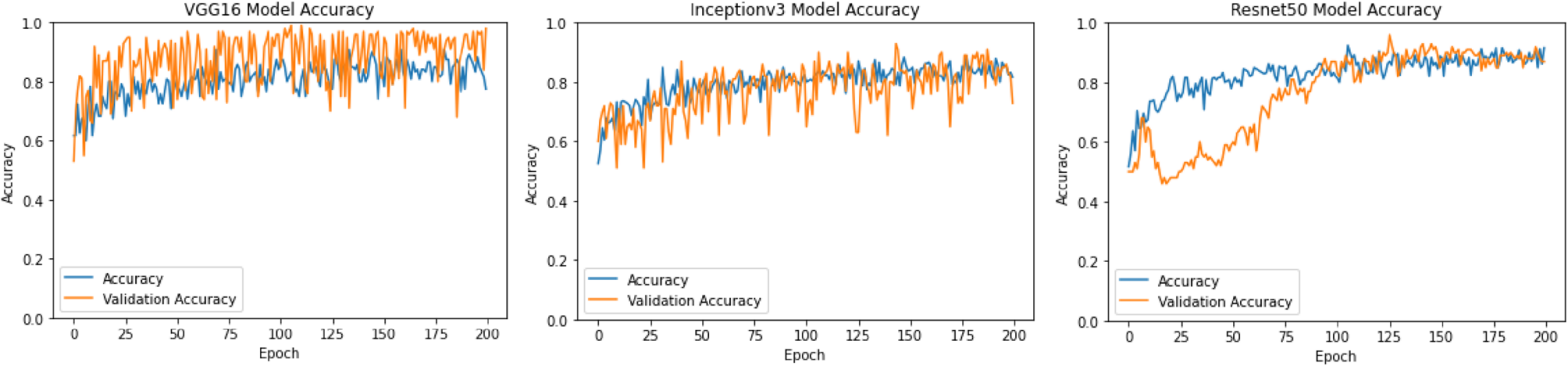
Performance evaluation of deep learning classifier on Brazilian Data-set.

To compare models’ performances on multi-class dataset (Hust19), and to show the proposed technique effectiveness, we calculated the overall precision, recall and accuracy. The results are illustrated in Figs. 6 and 8. The ensemble classifiers, Ada-Boost, Gradient-Boosting and Voting based classifiers have achieved accuracy of 81%, 99.0%, and 95.0% respectively. While on the contrary, deep learning-based classifiers, VGG16, Resnet50 and InceptionV3 results in 96%, 97.0%, and 97.9% accuracy. Figure 9 shows the results of deep learning classifiers (VGG16, InceptionV3, Resnet50) on Brazilian dataset.

The highest accuracy 99.6% on multi-class dataset is achieved by our proposed approach. Compared to the current deep learning based and ensemble classifiers, proposed ViT based approach has achieved better accuracy, suggesting that self-attention transformer using CT Scan images could be a reliable method in recognizing and detecting COVID-19 patients. Table 1 presents the accuracy of proposed approach, ensemble, and deep learning-based classifiers on binary and multi-class dataset. In Hust19 dataset, number of instances belonging to third class is very low as compared to other classes. Image variation in this dataset is also low and number of instances belonging to each variation are high that help the classifiers to achieve better accuracy.

**Table 1:** Accuracy of classifiers on binary and multi-class data-set.

S. no.	Approach	Test Accuracy on Brazilian Data-set (%)	Test Accuracy on Hust19 Data-set (%)
1	VGG16	85	96.0
2	Resnet50	90	97
3	InceptionV3	82	95.0
4	AdaBoost classifier	95	84
5	Voting based classifier	89	97
6	GradientBossting classifier	95	96.0
7	Proposed ViT based approach	98	97

### Comparison with existing studies

A comparison of proposed approach is performed with existing studies as well. There are several studies utilizing Brazilian and Hust19 dataset to diagnose the COVID-19 as illustrated in Table 2. In one of the research studies, a machine learning based algorithms was developed^33^ to diagnose COVID-19. Several machine learning models such as artificial neural networks, random forests, extra trees, gradient boosting and catboost were employed on Brazilian Dataset. All the models performed well, results in area under curve higher than achieved 92% and 82% sensitivity and specificity value respectively. In the proposed approach, tenfold cross validation was also performed. In another research study^34^, a voting-based approach was used for COVID-19 diagnosis. The proposed approach was applied on Brazilian dataset results in achieving accuracy and precision value of 87% and 99% respectively. In this approach, a cross dataset validation was also performed which illustrated that accuracy drops from 87 to 56%.

**Table 2:** Accuracy of classifiers on binary and multi-class data-set.

Refs.	Dataset	Approach	Accuracy (%)	AUROC (%)
^5^	BIMCV, NIH	ViT	86.9	0.92
^33^	Brazilian	Machine learning	87.66	0.9056
^34^	Brazilian	Voting based	87.66	0.906
^35^	Brazilian	xDNN	97.386	0.9736
^36^	COVID CT scans	CNN & ConvLSTM	99	–
^29^	Hust19	Deep learning		0.9946
^30^	COVID-19 CT Dataset	Transfer learning	83.6	0.946
^31^	Own Dataset	DeCovNet	90.16	0.9596
Proposed approach	Brazilian	Vision transformer	98	0.996
Proposed approach	Hust19	Vision transformer	99.7	0.997

Furthermore, xDNN was also applied in^35^ to diagnose the COVID-19 and Brazilian dataset, collected from different hospitals of Brazil was used for testing purpose. Moreover, xDNN classifier demonstrates good results in terms of explainable for detection of COVID-19 using CT slices. Furthermore, it also gives explanation using IF. THEN rules on actual CT scan images. The proposed approach^35^ achieved 97.38% accuracy. For detection of COVID-19, another approach^36^ utilized convolutional neural network and ConvLSTM. Approach was tested on two types of datasets which includes X-Ray and Brazilian CT scan images. In addition, pneumonia and COVID-19 image categories were classified for validation of approach^36^. Approach^36^ achieved an accuracy of 99% which reveals that it can be considered for quick screening of COVID-19. Table 2 demonstrate the results of existing classifiers for COVID-19 diagnosis. Table 2 demonstrate the results of existing classifiers for COVID-19 diagnosis. Our proposed vision-based transformer approach take 1 s per step in epochs for COVID-19 diagnosis.

### Cross-corpus data-set validation

**Figure 10:**
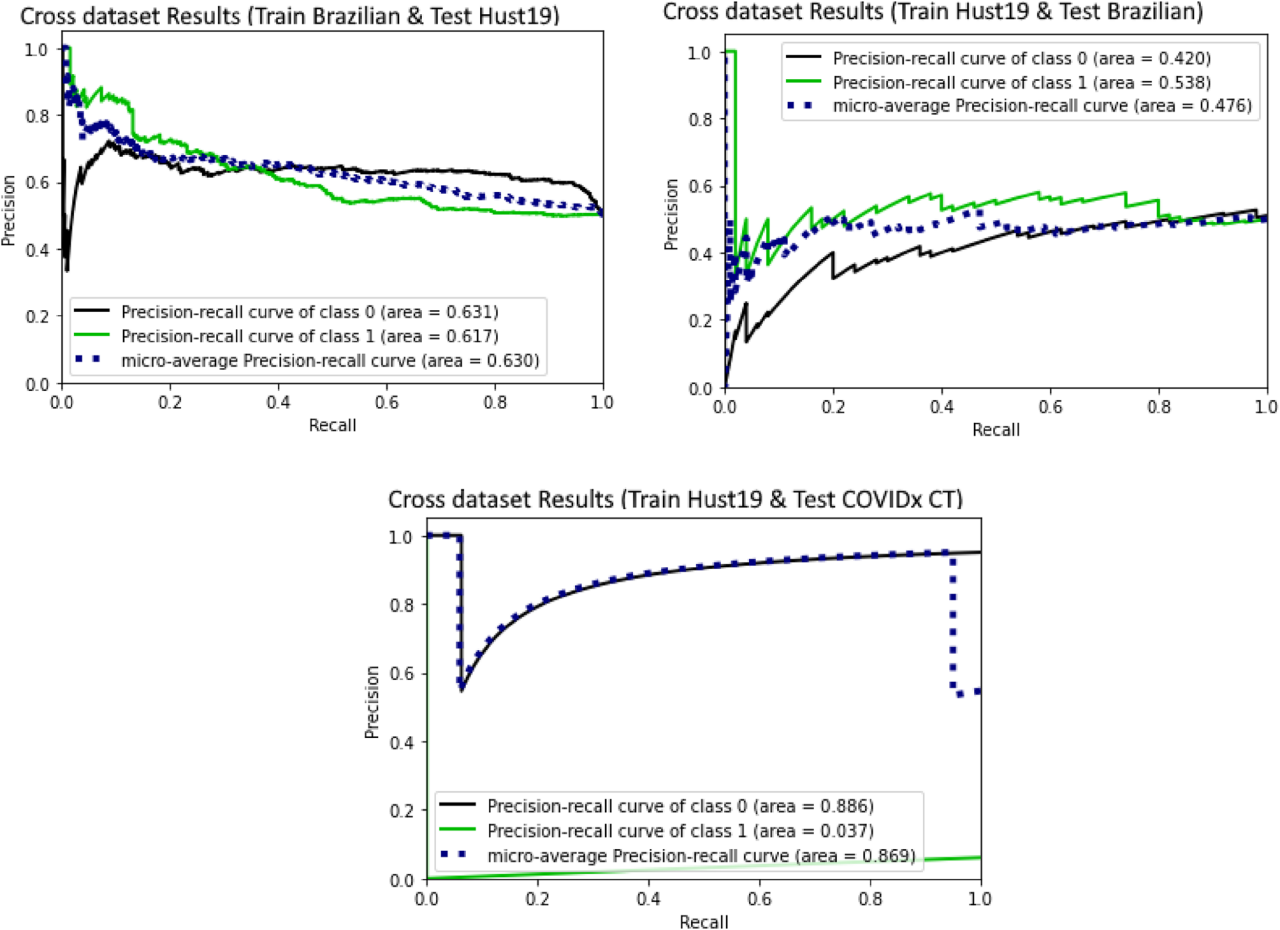
(**a**) Cross dataset Results (Train Brazilian & Test Hust19) (**b**) Cross dataset Results (Train Hust19 & Test Brazilian) (**c**) Cross dataset Results (Train Hust19 & Test COVIDx CT).

For this experiment, we examine the impact of training model on one dataset and testing it on another one. The Hust19 dataset is first used only for training and for testing the model, Brazilian dataset is used. We also evaluated another scenario such as using Brazilian dataset for training the model and Hust19 is used as a test set. The result of this scenario showed a decline in the model performance as can be seen in Fig. 10, and one of the possible reasons behind this behaviour can be variation in images. The model could find out the patterns of one image indicating COVID-19 existence, but these may not seem in another dataset. Training on Hust19 and testing Brazilian dataset showed worse results since Hust19 training set is not like Brazilian dataset. Since vision transformer model poorly generalize on small dataset that could be a reason in worse performance when cross corpus data-set validation is performed.

As we can see in Fig. 10c, precision and recall value of performing cross corpus between Hust19 and Brazilian dataset decreases rapidly although it was quite good in case of COVIDx CT dataset. Because Hust19 and COVIDx CT datasets are quite similar whereas Brazilian dataset was totally different from both datasets. Variation in images is high as compared to other datasets and number of images belonging to each variation are quite low. In case of training the model using Hust19 dataset and testing s performed using COVIDx dataset, proposed approach achieved accuracy of 94% which is higher than the existing studies^29–31^ having accuracy of 83.6% on Hust19 and 90% on their own dataset.

## Discussion

**Figure 11:**
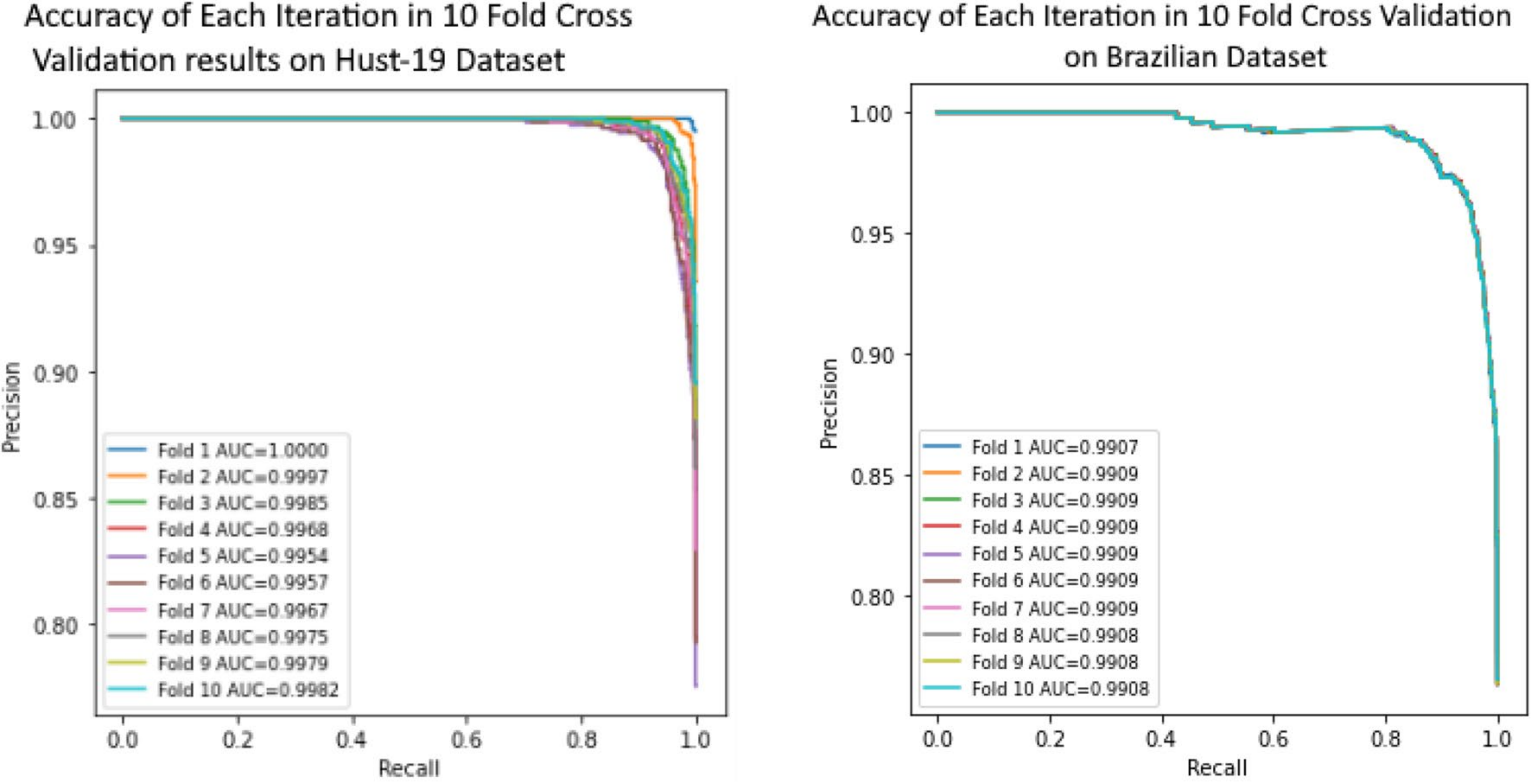
(**a**) 10 Fold Cross Validation results on Hust-19 Dataset (**b**) 10 Fold Cross Validation results on Brazilian Dataset.

Most of the classifiers performs well on binary dataset in comparison with multi-class dataset. However, classifiers accuracy on binary class dataset tends to be low as compared to multi-class data-set in proposed approach as depicted in Table 1. Binary dataset is heterogeneous and number of samples of CT scan images belonging to each variation are low. Whereas in multi-class dataset, image variation is quite low as compared to binary dataset and it comprises of a lot of images related to each variation. Thus, model performs better on multi-class dataset contrasted to binary class dataset. To evaluate the performance of proposed approach, tenfold cross validation is performed on binary and multiclass dataset as shown in Fig. 11a,b. Area under curve achieved in each fold (10 Fold Cross Validation) is represented in Fig. 11a,b.

## Conclusion

We have proposed a self-attention Transformer based diagnosis approach for the diagnosis of COVID-19 using 3D CT Slices. Results of the proposed approach are comparable to the state-of-the-art methods and has attained the highest accuracy on binary and multi-class datasets. Results validate the proposition that the proposed model aachieves good performance in the COVID-19 diagnosis on Brazilian dataset while outperforms the other techniques in case of the Hust19 dataset. In addition, we found that by applying ensemble, proposed and CNN based algorithms on HUST19 dataset achieved a much higher accuracy than Brazilian dataset. To the best of our knowledge, this is the first work to carry out such analysis based on transformer vision for the COVID diagnoses and we believe that this is a major contribution of our work. Cross corpus dataset validation is performed to evaluate the model performance using different datasets for testing and training and thus achieving higher performance. This is also unique contribution, as only existing study performing cross corpus validation dropped its accuracy by 25%^37^. The self-attention transformer-based approach is of paramount significance for the methods intent to diagnose the COVID-19 in CT scan images. Moreover, proposed transformer vision approach can predict the quantification of COVID-19 based on the pixel values in the long-range relation-based maps. This can provide the assistance to clinicians in decision making with respect to the assessment of the severity of the COVID-19.

## Data Availability

SARS-CoV-2 CT-scan dataset (Brazilian dataset) dataset is available on https://www.kaggle.com/plameneduardo/sarscov2-ctscan-dataset. Basic purpose of this dataset was to promote development and research of artificial intelligent methods that are able to determine person infected by SARS-CoV-2 using CT scans. In case of SARS-CoV-2 CT-scan dataset (Brazilian dataset) necessary IRB and/or ethics committee approvals was obtained. HUST19 Dataset is open source and is available on https://bioengineeringcommunity.nature.com/posts/hust-19-for-predicting-covid-19-clinical-outcomes. Hust19 was made available under a CC BY-NC 4.0 license. Hust19 was accumulated from lab of union hospital, Wuhan.
